# Are Mood and Anxiety Disorders and Alexithymia Associated with Endometriosis? A Preliminary Study

**DOI:** 10.1155/2014/786830

**Published:** 2014-06-22

**Authors:** Gabriele Cavaggioni, Claudia Lia, Serena Resta, Tatiana Antonielli, Pierluigi Benedetti Panici, Francesca Megiorni, Maria Grazia Porpora

**Affiliations:** ^1^Department of Neurology and Psychiatry, U.O.D. Psychotherapy, Sapienza University of Rome, Via Casal dei Pazzi 16, 00156 Rome, Italy; ^2^Department of Gynecology, Obstetrics and Urology, Sapienza University of Rome, Policlinico Umberto I, Viale del Policlinico 155, 00161 Rome, Italy; ^3^Department of Experimental Medicine, Sapienza University of Rome, Policlinico Umberto I, Viale Regina Elena 326, 00161 Rome, Italy

## Abstract

*Objective.* The aim of this preliminary study was to determine whether psychiatric disorders, psychopathological symptoms, and alexithymia are associated with endometriosis in an Italian population. *Study Design.* A preliminary study comprising 37 Italian patients with surgically confirmed endometriosis and 43 controls, without clinical and ultrasound signs of endometriosis, was carried out. Both patients and controls were evaluated for the presence/absence of psychiatric disorders, psychopathological symptoms, alexithymia, and pain symptoms (nonmenstrual pelvic pain, dysmenorrhea, and dyspareunia). *Results.* Statistically significant differences were found between cases and controls for prevalence of mood and anxiety disorders, malfunctioning on obsessive-compulsive subscale (*P* < 0.01) and depression subscale (*P* < 0.05) of the Symptom Checklist-90-Revisited (SCL-90-R), and higher alexithymia levels (*P* < 0.01). Patients with endometriosis-associated pain showed greater prevalence of psychiatric disorders compared to pain-free patients but that difference was not significant. Significant correlation was found between malfunctioning in some SCL-90-R dimensions and pelvic pain, dysmenorrhea, and dyspareunia scores at the visual analog score (VAS). *Conclusion.* Some psychopathological aspects, such as psychoemotional distress and alexithymia, are more frequent in women with endometriosis and might amplify pain symptoms in these patients.

## 1. Introduction

Endometriosis is a gynecological condition characterized by the presence of ectopic endometrial tissue (endometrial glands and stroma) outside of the uterus associated with pelvic pain and infertility [1]. The disease affects 6–10% of women in reproductive age, 50–60% of women and adolescent girls with pelvic pain, and more than 50% of infertile women [2, 3] and has a severe impact on the quality of life and work ability of employed women, representing a significant socioeconomic burden [4–7]. Endometriosis varies from a mild disease with only peritoneal lesions to a severe form involving both ovaries associated with infiltrating lesions and extensive adhesions. Women with endometriosis may have a range of pelvic and abdominal pain symptoms, including dysmenorrhea, dyspareunia, nonmenstrual (chronic) pelvic pain, pain at ovulation, dyschezia, and dysuria [8–10]. Endometriosis and its symptoms tend to recur after treatment [11]. Pain symptoms significantly vary among patients and do not always correlate with the severity of endometriosis [8–13] suggesting that other factors, such as psychological factors, altered stress response, and emotional factors, may influence the perception of pain [14]. Different studies have shown an increased prevalence of depressive symptoms and anxiety in women with endometriosis highlighting the importance of mood disorders in the perception of pain in these women [15–17].

The aim of the present study was to preliminarily evaluate the role of psychopathological symptoms, comorbid psychiatric disorders, and alexithymia in endometriosis patients compared with healthy women.

## 2. Materials and Methods

### 2.1. Patients and Controls

Thirty-seven Italian Caucasian women with endometriosis (mean age 35 ± 7.6 years), ranging from unskilled workers to university graduates, were included in the study. Patients were recruited from the Department of Gynecology and Obstetrics, “Sapienza” University of Rome, Italy. Diagnosis of endometriosis was achieved by laparoscopy and histologic analysis. Twenty-one of them (56.8%) had ovarian endometrioma, while sixteen (43.2%) had ovarian endometrioma and peritoneal endometriosis. No other comorbid physical conditions were present. As healthy controls, 43 women from the same ethnic area, referred to the gynecological clinic for a gynecological control, were enrolled, mean age of 34.9 ± 10.1 years and medical history, vaginal pelvic examination, and ultrasound (US) imaging with color Doppler flow evaluation negative for endometriosis. The ethical committee of the “Sapienza” University of Rome approved the study protocol and all subjects provided their informed consent.

### 2.2. Symptoms and Pain Assessment

Patients were evaluated for the presence/absence of symptoms (nonmenstrual pelvic pain, dysmenorrhea, and dyspareunia). Pain intensity was assessed by a 10-point visual analogue scale (VAS) with 0 representing no pain and 10 representing the worst pain [18].

### 2.3. Psychometric Testing

Assessment of psychiatric disorders was performed using the Structured Clinical Interview for DSM IV Axis I Disorders Clinical Version (SCID-I cv) [19] for each subject, after a clinical interview performed by a psychiatrist experienced in the use of this instrument.

The presence of psychopathological symptoms was investigated using the Symptom Checklist-90-Revised (SCL-90-R), a 90-item self-report instrument that has been designed to evaluate a broad range of psychological problems and symptoms of psychopathology [20]. We considered 9 subscales: somatization (SOM), obsessive-compulsive (OC), interpersonal sensitivity (SENS), depression (DEP), anxiety (ANX), hostility (HOS), phobic anxiety (PHOB), paranoid ideation (PAR), and psychoticism (PSY) (24). A score > 60 T points was indicative of malfunctioning.

The Toronto Alexithymia Scale (TAS-20) was used to assess alexithymia. It consists of 20 self-report items and has 3 subscales: Difficulty Describing Feelings (F1), Difficulty Identifying Feelings (F2), and Externally Oriented Thinking (F3). A TAS-20 total score ≥ 61 is diagnostic for alexithymia [21].

### 2.4. Statistical Analysis

Basic statistical analyses were performed with the Statistical Product and Service Solutions software (SPSS) version 17.0 WIN program (SPSS, Chicago, IL). The *χ* ^2^ or Fisher's exact tests were used to compare differences between categorical variables, whereas those between continuous variables were determined by Student's* t*-test or Mann-Whitney* U* test. Spearman's rho correlation coefficient was applied to analyze nonparametric correlations. A *P* value < 0.05 was considered statistically significant. Test results from the nine SCL-90-R subscales were processed by ARC90 v1.2.1 software [22].

## 3. Results

Thirty-seven patients and 43 controls underwent psychiatric evaluation. The two groups were homogeneous for age (patients mean age 35.0 ± 7.6, controls mean age 34.9 ± 10.1;* t* = −0.034, *P* = 0.973), educational status (*χ* ^2^ = 5.335; *P* = 0.149), marital status (*χ* ^2^ = 6.713; *P* = 0.082), and occupational status (*χ* ^2^ = 5.915; *P* = 0.657). Pain evaluation showed higher dyspareunia and pelvic pain VAS mean scores in patients with endometriosis than in controls, while no differences were observed for dysmenorrhea VAS mean scores (Table 1).

The frequency of psychiatric disorders was 54.0% in patients and 18.6% in controls (*χ* ^2^ = 10.985; *P* = 0.001); the relative percentages of each disorder are shown in Table 2. No significant difference was observed between the two groups regarding the presence of a specific psychiatric disorder; while grouping the disorders in categories the frequencies of mood and anxiety disorders were significantly higher in patients than in controls (Table 3). No significant difference in psychiatric comorbidity between pelvic pain, dysmenorrhea, and dyspareunia VAS subgroups (VAS = 0–5 and VAS = 6–10) in the overall sample was found (data not shown). However, considering only the patients group a statistically significant difference was observed in the frequency of mood and anxiety disorders between women with pelvic pain (10 subjects) and women without pelvic pain (8 subjects) (70.0% and 37.5%, resp.; Fisher's exact test, *P* = 0.0342).

Regarding the SCL-90-R and the TAS-20 instruments, four tests in the patient group were discarded because of being invalid.  Table 4 shows the frequencies distribution in subjects with scores > cut-off. No statistically significant difference in the frequency distribution between cases and controls was observed, except for obsessive-compulsive (*χ* ^2^ = 15.005; *P* < 0.01) and depression subscales (*χ* ^2^ = 4.035; *P* = 0.045). Mean ranks comparison performed with Mann-Whitney* U* test showed statistically significant differences between patients and controls group in relation to TAS-20 total score (*P* < 0.01), Difficulty Identifying Feeling (*P* < 0.05), and Externally Oriented Thinking (*P* < 0.01). Comparing SCL-90-R and TAS-20 results in the presence/absence of comorbid psychiatric disorder groups, no differences were found in frequency distribution and mean ranks comparison. In the patients group, the analysis of VAS mean scores (chronic pelvic pain, dysmenorrhea, and dyspareunia) in absence/presence of comorbid psychiatric disorders (also specifically for depressive spectrum disorders), malfunctioning in SCL-90-R subscales, and alexithymia in TAS-20 showed that significant differences were present for chronic pelvic pain and dysmenorrhea (Student's* t*-test; Table 5). Statistically significant correlation was observed between chronic pelvic pain and TAS-20 total score and between dyspareunia, Difficulty Describing Feelings, and Difficulty Identifying Feelings (Table 6). The analysis of VAS mean scores did not show statistically significant results for any of the investigated variables in the control group.

Finally, the correlation between the type of endometriosis (ovarian endometrioma or ovarian endometrioma and peritoneal endometriosis) and psychopathological symptoms or VAS mean scores did not show significant results.

## 4. Discussion

This preliminary study aimed to explore the possible role of psychiatric comorbidity, psychopathological symptoms, and alexithymia in endometriosis and their correlation with pain symptoms.

Even if in our study no statistically significant differences were observed between cases and controls regarding frequency of a specific Axis I DSM IV-TR diagnosis, the prevalence of psychiatric disorders was statistically higher in patients than in controls and a statistically significant difference was also found for mood and anxiety disorders and malfunctioning on obsessive-compulsive and depression SCL-90 subscales.

In the present study psychiatric comorbidity of patients with endometriosis was lower than that reported by previous studies [15–17]. Although not statistically significant, the higher prevalence of mood and anxiety disorders observed in patients with chronic pelvic pain compared with those with no pain confirmed previous findings [15, 23, 24]; however our observations were in contrast with some studies showing no difference between these subgroups in terms of frequencies of depression and anxiety disorders [25] or mental health status [26]. Since the small sample sizes (10 versus 8 subjects) of our subgroups might be a strong limitation, any outcome should be cautiously considered.

Data analysis revealed some interesting results: (1) patients with endometriosis showed higher scores on TAS-20 total score, Difficulty Identifying Feeling, and Externally Oriented Thinking (that indicates an inadequate introspective ability) than controls; (2) among patients with endometriosis, greater chronic pelvic pain VAS score was observed in patients with alexithymia (TAS-20 total score ≥ 61); (3) a statistically significant correlation was found between TAS-20 total score, Difficulty Describing Feelings, and Difficulty Identifying Feelings and chronic pelvic pain and dyspareunia VAS scores in patients group. These findings could suggest that the difficulty to recognize feelings and emotions in endometriosis patients may facilitate somatization, that is, the expression of psychological problems on a somatic level. Similar assumptions could be done also in relation to malfunctioning in somatization and sensitivity SCL-90-R subscales, correlated with higher chronic pelvic pain and dysmenorrhea (only somatization) VAS scores. Finally, the observed greater chronic pelvic pain VAS scores in patients with malfunctioning in anxiety and obsessive-compulsive SCL-90-R subscales were in line with what was shown by previous studies regarding the possible association with the presence of psychiatric comorbidity (above all of anxiety spectrum disorders) and pelvic pain in patients with endometriosis. In addition, our study highlighted the prevalence of a greater dysmenorrhea VAS score in patients with comorbid psychiatric disorders and those with malfunctioning in obsessive-compulsive SCL-90-R subscales, while no significant correlations were found between the type of endometriosis and the presence of psychopathological symptoms or pain symptoms.

## 5. Conclusions

Even if in our study we could not find a specific association with certain psychopathological features and the presence of endometriosis, however the subgroups of patients with chronic pelvic pain, dysmenorrhea, and dyspareunia were characterized by the presence of a specific grade of psychopathology that could play a role in pain perception and reaction.

The small sample size of this preliminary study certainly represented a limitation and did not allow us to determine a specific causality relation between some psychological features and endometriosis onset or pain perception or to exactly identify specific risk factors. Therefore, these preliminary findings need to be confirmed by further investigations and increasing the sample size.

However, this study highlighted some significant characteristics. Indeed, these results suggested that some mood and psychiatric characteristics such as mood and anxiety disorders, higher alexithymia levels, and malfunctioning on obsessive-compulsive and depression dimension are more frequent in women with endometriosis than in general population. Moreover, these psychopathological conditions could be correlated with moderate-severe pain symptoms and could influence pain perception in endometriosis patients.

## Figures and Tables

**Table 1:** Pain intensity evaluation.

	Dyspareunia VAS scores		Dysmenorrhea VAS scores		Pelvic pain VAS scores	
	Mean ± SD	*P*	Mean ± SD	*P*	Mean ± SD	*P*
Patients	2.5 ± 3.2	0.003	5.3 ± 3.8	0.365	2.1 ± 3.6	<0.001
Controls	0.7 ± 2.1		4.6 ± 3.5		0.02 ± 0.2	

**Table 2:** DSM IV-TR Axis I disorders.

Diagnosis	Patients		Controls	
	*n*	%	*n*	%
Psychotic disorder NOS	1	(2.7%)	0	(/)
Dysthymic disorder	3	(8.1%)	1	(2.3%)
Major depressive disorder	2	(5.4%)	3	(7.0%)
Depressive disorder NOS	1	(2.7%)	0	(/)
Bipolar II disorder	1	(2.7%)	0	(/)
Generalized anxiety disorder	1	(2.7%)	1	(2.3%)
Panic disorder without agoraphobia	1	(2.7%)	0	(/)
Specific phobia	3	(8.1%)	2	(4.7%)
Social phobia	1	(2.7%)	0	(/)
Obsessive-compulsive disorder	1	(2.7%)	0	(/)
Anxiety disorder NOS	4	(10.8%)	0	(/)
Eating disorder NOS	0	(/)	1	(2.3%)
Undifferentiated somatoform disorder	1	(2.7%)	0	(/)
No diagnosis or condition on Axis I	17	(46.0%)	35	(81.4%)

(*χ* ^2^ = 19.289; *P* = 0.114).

**Table 3:** DSM IV-TR Axis I disorders.

Diagnosis	Patients		Controls	
	*n*	%	*n*	%
Psychotic disorders	1	(2.7%)	0	(/)
Mood disorders	7	(18.9%)	4	(9.3%)
Anxiety disorders	11	(29.7%)	3	(7.0%)
Eating disorders	0	(/)	1	(2.3%)
Somatoform disorders	1	(2.7%)	0	(/)
No diagnosis or condition on Axis I	17	(46.0%)	35	(81.4%)

(*χ* ^2^ = 14.251; *P* = 0.014).

**Table 4:** Frequencies of scores > cut-offs in SCL-90-R subscales and TAS-20 total score.

	Scores > cut-offs		*χ* ^2^	*P*
	Patients	Controls		
Somatization	11 (33.3%)	11 (25.6%)	0.546	0.460
Obsessive-compulsive	10 (30.3%)	0 (0%)	15.005	0.000∗∗
Interpersonal sensitivity	13 (39.4%)	10 (23.3%)	2.304	0.129
Depression	13 (39.4%)	8 (18.6%)	4.035	0.045∗
Anxiety	8 (24.2%)	6 (14.0%)	1.315	0.251
Hostility	5 (15.2%)	10 (23.3%)	0.774	0.379
Phobic anxiety	1 (3.0%)	4 (9.3%)	1.195	0.274
Paranoid ideation	9 (27.3%)	13 (30.2%)	0.080	0.778
Psychoticism	5 (15.2%)	5 (11.6%)	0.203	0.652
TAS-20 total score	6 (18.2%)	5 (11.6%)	0.353	0.552

*Frequencies difference is significant at the 0.05 level.

∗∗Frequencies difference is significant at the 0.01 level.

**Table 5:** Pelvic pain and dysmenorrhea VAS mean scores comparison in relation to presence of psychiatric disorders, malfunctioning on SCL-90 subscales, and alexithymia (TAS-20 total score) in endometriosis patients.

		Pelvic pain		Dysmenorrhea	
		Mean ± SD	*P*	Mean ± SD	*P*
Comorbid psychiatric disorder	absent	1.3 ± 2.8	0.286	3.9 ± 4.1	0.049∗
	present	2.6 ± 4.1		6.4 ± 3.3	

SOM	<60 T points	0.68 ± 2.2	0.003∗∗	4.1 ± 3.7	0.011∗
	>60 T points	4.4 ± 4.4		7.5 ± 4.0	

OC	<60 T points	0.65 ± 2.2	0.001∗∗	4.4 ± 4.7	0.048∗
	>60 T points	4.8 ± 4.4		7.2 ± 3.4	

SENS	<60 T points	0.90 ± 2.8	0.038∗	5.2 ± 3.6	0.865
	>60 T points	3.4 ± 4.0		5.4 ± 4.2	

ANX	<60 T points	0.96 ± 2.7	0.004∗∗	4.6 ± 3.8	0.107
	>60 T points	4.9 ± 2.3		7.1 ± 3.4	

TAS-20	≤60	1.1 ± 2.7	0.004∗∗	4.7 ± 3.7	0.376
	≥61	5.5 ± 4.5		6.5 ± 4.1	

*Means difference is significant at the 0.05 level.

∗∗Means difference is significant at the 0.01 level.

**Table 6:** Correlation between pelvic pain and dyspareunia VAS scores and TAS-20, Difficulty Describing Feelings (F1), and Difficulty Identifying Feeling (F2) scores in endometriosis patients. Spearman's rho correlations.

		TAS-20 total score	F1	F2
Pelvic pain	Correlation coefficient	0.359∗	0.247	0.251
	Sig. (2-tailed)	0.040	0.165	0.158
	*N*	33	33	33

Dyspareunia	Correlation coefficient	0.104	0.345∗	−0.357∗
	Sig. (2-tailed)	0.565	0.049	0.041
	*N*	33	33	33

*Correlation is significant at the 0.05 level.
